# Range‐wide persistence of the endangered arroyo toad (*Anaxyrus californicus*) for 20+ years following a prolonged drought

**DOI:** 10.1002/ece3.8796

**Published:** 2022-04-19

**Authors:** Cynthia J. Hitchcock, Elizabeth A. Gallegos, Adam R. Backlin, Russell Barabe, Peter H. Bloom, Kimberly Boss, Cheryl S. Brehme, Christopher W. Brown, Denise R. Clark, Elizabeth R. Clark, Kevin Cooper, Julie Donnell, Edward Ervin, Peter Famolaro, Kim M. Guilliam, Jacquelyn J. Hancock, Nicholas Hess, Steven Howard, Valerie Hubbartt, Patrick Lieske, Robert Lovich, Tritia Matsuda, Katherin Meyer‐Wilkins, Kamarul Muri, Barry Nerhus, Jeff Nordland, Brock Ortega, Robert Packard, Ruben Ramirez, Sam C. Stewart, Samuel Sweet, Manna Warburton, Jeffrey Wells, Ryan Winkleman, Kirsten Winter, Brian Zitt, Robert N. Fisher

**Affiliations:** ^1^ 233286 U.S. Geological Survey, Western Ecological Research Center Santa Ana California USA; ^2^ 66621 California Department of Fish and Wildlife San Diego California USA; ^3^ Bloom Biological, Inc. Santa Ana California USA; ^4^ 533817 San Bernardino National Forest Idyllwild California USA; ^5^ U.S. Geological Survey, Western Ecological Research Center San Diego California USA; ^6^ 177326 US Army Garrison Fort Hunter Liggett Fort Hunter Liggett California USA; ^7^ 390821 Los Padres National Forest Santa Barbara California USA; ^8^ San Bernardino National Forest Fawnskin California USA; ^9^ Merkel and Associates, Inc. San Diego California USA; ^10^ Sweetwater Authority Chula Vista California USA; ^11^ Independent Researcher Glendale California USA; ^12^ R2 Resource Consultants, Inc. Ventura California USA; ^13^ Los Padres National Forest Santa Barbara California USA; ^14^ 8381 Naval Facilities Engineering Command Southwest San Diego California USA; ^15^ Independent Researcher Bellevue Idaho USA; ^16^ Dudek Encinitas California USA; ^17^ Endemic Environmental Huntington Beach California USA; ^18^ Southwest Field Herping Association San Diego California USA; ^19^ Western Riverside County MSHCP Biological Monitoring Program Riverside California USA; ^20^ Cadre Environmental Carlsbad California USA; ^21^ Southwest Aquatic and Terrestrial Biology Long Beach California USA; ^22^ 8786 UC Santa Barbara Santa Barbara California USA; ^23^ ICF Eureka California USA; ^24^ Cleveland National Forest San Diego California USA; ^25^ Independent Researcher Santa Ana California USA; ^26^ 263260 ECORP Consulting Inc. Santa Ana California USA

**Keywords:** amphibian decline, California, climate change, endangered species, riparian habitat

## Abstract

Prolonged drought due to climate change has negatively impacted amphibians in southern California, U.S.A. Due to the severity and length of the current drought, agencies and researchers had growing concern for the persistence of the arroyo toad (*Anaxyrus californicus*), an endangered endemic amphibian in this region. Range‐wide surveys for this species had not been conducted for at least 20 years. In 2017–2020, we conducted collaborative surveys for arroyo toads at historical locations. We surveyed 88 of the 115 total sites having historical records and confirmed that the arroyo toad is currently extant in at least 61 of 88 sites and 20 of 25 historically occupied watersheds. We did not detect toads at almost a third of the surveyed sites but did detect toads at 18 of 19 specific sites delineated in the 1999 Recovery Plan to meet one of four downlisting criteria. Arroyo toads are estimated to live 7–8 years, making populations susceptible to prolonged drought. Drought is estimated to increase in frequency and duration with climate change. Mitigation strategies for drought impacts, invasive aquatic species, altered flow regimes, and other anthropogenic effects could be the most beneficial strategies for toad conservation and may also provide simultaneous benefits to several other native species that share the same habitat.

## Graphical abstract

U.S. range‐wide surveys for arroyo toads were conducted at 100% of the historical watersheds and ~76% of the historical sites following prolonged drought. Arroyo toads were detected at most sites delineated in the 1999 Recovery Plan for a downlisting consideration, yet toads could not be detected at 31% of sites surveyed indicating further declines. These are the most current and comprehensive data on number of extant arroyo toad populations since 1999 and can be used as a baseline for reevaluation of the Recovery Plan.

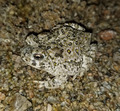

## 1 INTRODUCTION

Climate can be a major driver of amphibian health and persistence, and survival strategies influenced by climate change could contribute to population extinctions (Bucciarelli et al., 2020). For example, global warming and severe drought decrease body size and body condition in many amphibian species, which consequently decreases survivorship and fecundity (Caruso et al., 2014; Cayuela et al., 2016; Reading, 2007; Stanley et al., 2020). Climate‐induced habitat changes, such as decreased availability of surface water for breeding, can also cause amphibian populations to decline (Miller et al., 2018). Decline and extinction rates from climate change could be exacerbated by anomalous catastrophic events and anthropogenic threats. Ultimately, the rate of environmental change resulting from warming and drought, along with compounding effects of anthropogenic threats may exceed the rate of adaptation or resiliency for many amphibians. This is especially a concern in southern California where freshwater is extremely limited to begin with, and drought might cause limited surface water to dry up in some years.

California, U.S.A., has experienced notable changes in climate over the last several decades. For example, LaDochy et al. (2007) report that the mean temperature in the state has risen by approximately 0.99°C since 1950, and Goss et al. (2020) report a similar increase in California's mean temperature since 1980. The southern California region has experienced numerous extreme wildfire events (Goss et al., 2020; Keeley et al., 2009; Nauslar et al., 2018; Tracey et al., 2017) and unprecedented drought over the last decade (Fisher et al., 2018; Griffin & Anchukaitis, 2014; Swain et al., 2014). In addition, the rapid growth rate of the human population and urban development in southern California (Kindlmann et al., 2005) conflicts with preserving habitat for the region's extraordinary biodiversity, which holds a high level of endemism resulting in part from the region's diverse geomorphology and climate (Dobson et al., 1997; Howard et al., 2013; Myers et al., 2000; Wilson, 1992). The effects of climate change, wildfire, drought, disease, invasive species, and other anthropogenic impacts are threatening amphibian persistence in southern California and adjacent northern Baja California (Bucciarelli et al., 2020; Diffenbaugh et al., 2015; Griffin & Anchukaitis, 2014; Jones et al., 2017; Miller et al., 2012; Peralta‐Garcia et al., 2016; Richmond et al., 2021; Russell et al., 2019).

**FIGURE 1:**
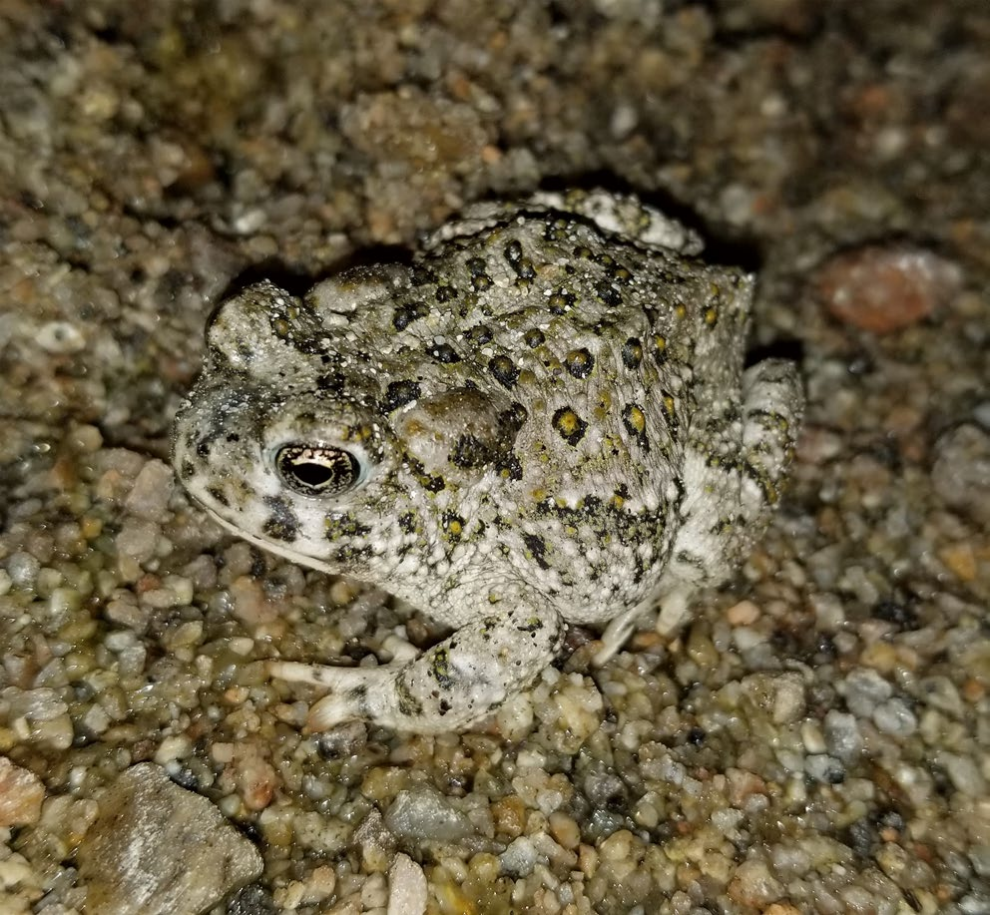
Arroyo toad (*Anaxyrus californicus*)

The arroyo toad (*Anaxyrus californicus*; Figure 1) is endemic to southwestern California and northern Baja California and is federally listed as endangered in the United States and by México (Hammerson & Santos‐Barrera, 2004; Poder Ejecutivo Federal, 2008; Sweet & Sullivan, 2005; Thomson et al., 2016; USFWS, 1994). This species was listed under the Endangered Species Act in 1994 after reported declines from approximately 75% of formerly occupied habitat across its range in California (Jennings & Hayes, 1994; Sweet, 1992; USFWS, 1994). Its status was retained upon reevaluation in 2014 (USFWS, 2015). Anthropogenic threats identified by various authors include off‐highway vehicle (OHV) use, dam/hydrological operations, disease, and invasive species (Ervin et al., 2006; Funk et al., 2014; Madden‐Smith et al., 2003; Miller et al., 2012; Ramirez, 2003; Robeson, 2015; Sweet, 1992). In the United States, the arroyo toad historically occupied 25 watersheds along mostly coastal and a few desert drainages from Monterey County to San Diego County (Ervin et al., 2013; USFWS, 2015). The 1999 USFWS Recovery Plan for the arroyo toad lists 20 (but actually 19 due to a misidentification; see Ervin et al., 2013) populations at specific locations that must be self‐sustaining for a downlisting consideration (USFWS, 1999, pp. 75–76). According to the Recovery Plan, self‐sustaining populations are defined as “having successful recruitment equal to 20% or more of the average number of breeding individuals in seven of ten years of average to above‐average rainfall amounts with normal rainfall patterns” (USFWS, 1999, p. 76).

The arroyo toad is a habitat specialist, requiring low‐gradient intermittent streams and rivers with sandy terraces and banks, as well as gravel and sand bars (Cunningham, 1962; Sweet, 1992; Sweet & Sullivan, 2005). Reproduction is dependent upon the availability of shallow and slow‐moving streams typical of a natural flood‐disturbed environment in which breeding, egg laying, and larval development occur (Sweet, 1992; Sweet & Sullivan, 2005; Thomson et al., 2016; USFWS, 1999). These habitat features are largely dependent on natural hydrological cycles and scouring events (Jennings & Hayes, 1994). A recent study on longevity estimates that this species lives approximately 7–8 years on average (Fisher et al., 2018). The drought in southern California peaked in 2012–2016 (Diffenbaugh et al., 2015; Griffin & Anchukaitis, 2014) and has continued through 2022 despite some occasional wet years (https://www.ncdc.noaa.gov/cag/divisional/time‐series/0406/pcp/12/12/2000‐2022?trend=true&trend_base=10&begtrendyear=2000&endtrendyear=2022). Given this prolonged period of drought, there has been growing concern that the number of consecutive years of drought may have surpassed the lifespan of the species (Fisher et al., 2018), and recruitment may have been severely diminished due to lack of surface water (especially in ephemeral watersheds), resulting in possible population declines and local extirpations. Additionally, evidence of direct mortality of toads due to drought was reported during a telemetry study that included observations of desiccated toads found under the sand in which they had burrowed (Gallegos, 2011–2013, 2016 unpublished data). These concerns prompted collaborative, range‐wide surveys for the arroyo toad in 2017 that continued with several additional surveys through 2020.

We investigated population status by surveying known historical arroyo toad locations within the United States and compared the locations where toads were detected/not detected to locations where they were extant in 1999 (the time the recovery plan was written; USFWS, 1999) and in 2014 (the time of the last reevaluation of their status; USFWS, 2014, 2015). To cover the extent of the historical locations within the United States, we formed a collaboration of researchers to comprehensively survey as many historical sites as possible from Monterey County to San Diego County from 2017 to 2020 and combined our detection/non‐detection findings.

### 1.1 Study area

**FIGURE 2:**
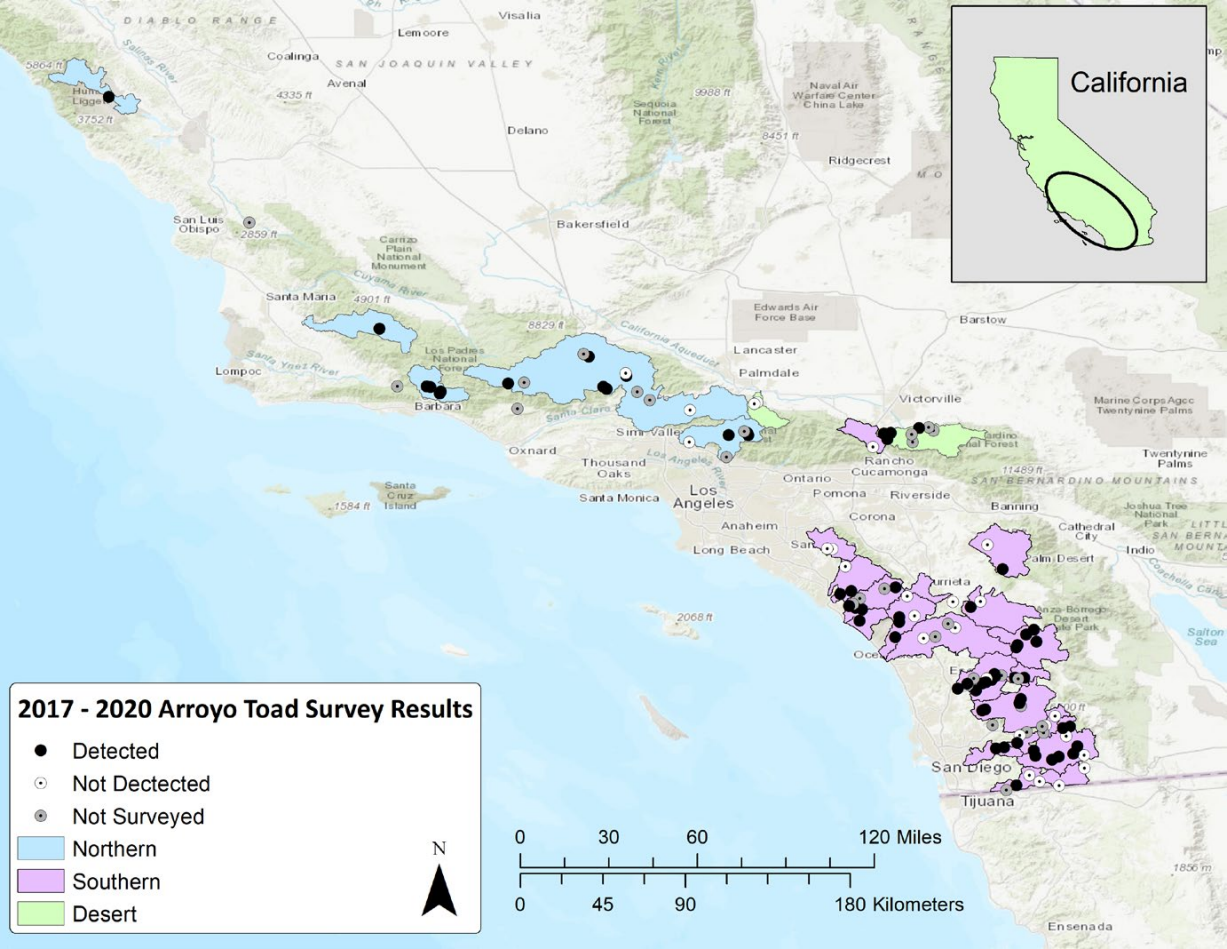
Of the 115 known sites in the United States, 88 were surveyed as part of this effort and arroyo toads were detected at 61 (see also Appendix)

Our study area included all the watersheds in the United States within the known range of the arroyo toad that were delineated in the 1999 Recovery Plan (USFWS, 1999). A few locations have been updated and revised from the original Recovery Plan to account for corrections made after publication (Ervin et al., 2013; USFWS, 2014). The revised total includes 25 watersheds spanning Monterey to San Diego counties. Multiple sites within these watersheds were surveyed to determine presence (Figure 2). All sites described in the 1999 Recovery Plan, 2014 Species Report (USFWS, 2014), or other literature and databases were treated as separate locations within shared watersheds, and all had arroyo toads historically. Rainfall and temperatures are highly variable throughout the broad geographic range of this species, with several different ecoregions inhabited, including both desert and coastal drainages. According to the National Oceanic and Atmospheric Administration (NOAA), annual rainfall since 1950 varied from 50 to 325 millimeters (mm) in the desert basins (https://www.ncdc.noaa.gov/cag/divisional/time‐series/0407/pcp/12/12/1950‐2022?trend=true&trend_base=10&begtrendyear=1950 &endtrendyear=2022) and 135–900 mm in the south coast drainages (https://www.ncdc.noaa.gov/cag/divisional/time‐series/0406/pcp/12/12/1950‐2022?trend=true&trend_base=10&begtrendyear=1950&endtrendyear=2022); in general, the southern California climate is hot and dry in summer with cooler temperatures and low to moderate rainfall in winter. Air temperatures at many arroyo toad sites can briefly drop below freezing at times during winter but can also reach in excess of 40°C in summer.

## 2 METHODS

We collaborated as 37 partners from 19 various state and government agencies, consulting groups, universities, and independent researchers, to survey for arroyo toads at historical locations. We compiled range‐wide comprehensive and current data for the detection/non‐detection of the species at as many sites as possible that were listed in the 1999 Recovery Plan and other literature or databases (mostly the USFWS 2014 Species Report). Most of our surveys were conducted during 2017, but several sites initially skipped for logistical reasons were surveyed 2018–2020. Incidentally, the winter of 2018–2019 had more rainfall compared to the surrounding years; therefore, toads were expected to be more easily detectable during that year. Location descriptions and Global Positioning System (GPS) coordinates from the Recovery Plan, grey literature, and from biologists who had been to the sites were used to determine the precise multiple locations to survey within the 25 watersheds. Because arroyo toads have been documented as having an average dispersal distance of ~3 km (USFWS, 1999), long swaths of habitat were surveyed within each documented location to account for movement of arroyo toads up and down waterways even if some of the habitat was marginal. A location within a watershed was regarded as a “site” if it had historical records of arroyo toads described for that specific location within the watershed. We also considered the ~3 km average dispersal distance (USFWS, 1999) and any geographic barriers (i.e., mountains, urban development) to establish which locations we could regard as being a single “site” versus more than one “site” within a watershed. Our team of collaborators developed a spreadsheet of arroyo toad sites to survey, categorized by watershed and recovery unit. The spreadsheet included the following: (1) a list of all known arroyo toad historical sites based on literature (mostly from the 1999 Recovery Plan and USFWS 2014 Species Report), and (2) fields for participants to provide date surveyed, specific location, and age class observed. Collaborators throughout southern California conducted surveys at as many locations as possible, mostly according to their proximity to nearby sites. Participants conducted daytime and/or night surveys during the breeding and active season of the toad (generally April–July depending on elevation, latitude, and local climate). Surveyors walked the creeks at historical locations surveying visually and dip‐netting for tadpoles during the day. There was no minimum or maximum number of linear meters walked; the presence of suitable (and even marginal) arroyo toad habitat dictated the length of creek surveyed. If no toads or larvae were detected during the day, most surveys were continued at nighttime along the same length of creek and in the same manner by looking but also listening for calling adults. One survey (day or day/night) per site was made, although occasionally different participants happened to overlap the same site. Data from all participants were compiled and number of locations where toads were detected/not detected were compared to number of locations where toads were recorded as extant in the 1999 Recovery Plan. Years that toads were last documented from all sites were also compiled (Appendix).

We also examined weather data, reports, unpublished data, and gray literature from past surveys conducted by U.S. Geological Survey (USGS), U.S. Forest Service (USFS), U.S. Fish and Wildlife Service (USFWS), and other partners to assess whether any anomalous events may have affected population presence or detectability (i.e., major local weather events or anthropogenic changes to the habitat) at any sites. To gain perspective on climate change in the region, we compiled literature and online climate data (https://www.ncdc.noaa.gov/cag/divisional/time‐series/0406/tavg/12/12/1950‐2022?trend=true&trend_base=10&begtrendyear=1950&endtrendyear=2022, and https://www.ncdc.noaa.gov/cag/divisional/time‐series/0406/pcp/12/12/1950‐2022?trend=true&trend_base=10&begtrendyear=1950&endtrendyear=2022, accessed March 2022; NOAA, 2022) and plotted average annual temperatures and precipitation from 1950–2020 for California's South Coast Drainage. Our precipitation and temperature profiles were produced from data on the NOAA website by selecting the “divisional” tab at the top, then “time series,” then parameters from the drop‐down menus for temperature or precipitation, annual average, bounding years (1950–2021), “state” (California) and “division” (south coast drainage). Precipitation measurements were converted to millimeters and temperature was converted to degrees Celsius. These data were graphed in Microsoft Excel™ to show the difference in temperature and precipitation from the mean over time.

## 3 RESULTS

Of the more than 70 individuals asked to participate in surveys, we received data and input from 37 researchers and citizen scientists. Collectively, we were able to survey within all 25 (~100%) historical watersheds and at 88 of the 115 (~76.5%) individual sites assembled mainly from the 1999 Recovery Plan and USFWS 2014 Species Report. Due to logistical and time constraints, 27 sites were not surveyed. At the watershed scale, arroyo toads were detected in 20 of 25 (80%) watersheds surveyed (Table 1). At the site scale, arroyo toads were detected at 61 of the 88 (~69%) sites surveyed (Figure 2; Appendix). Diurnal surveys detected toads at 52 of 61 extant sites. Nocturnal surveys detected toads at the remaining nine extant locations. Our diurnal surveys detected toads during ~85% of the total surveys conducted.

**TABLE 1:** Summary of detection/non‐detection results for the 25 watersheds historically occupied by arroyo toads in the U.S. (USFWS, 1999)

Recovery unit	# Watersheds in recovery unit	Watershed names	^a^Detected Yes or No
Northern	5	Los Angeles River Basin	Y
		Salinas River Basin	Y
		Santa Clara River Basin	Y
		Santa Maria River Basin	Y
		Santa Ynez River Basin	Y
Southern	18	Cottonwood Creek Basin (lower)	Y
		Cottonwood Creek Basin (upper)	Y
		Murrieta Creek Basin	N
		San Diego River Basin (upper)	Y
		San Jacinto River Basin	Y
		San Juan Creek Basin	Y
		San Luis Rey River Basin (lower and middle)	N
		San Luis Rey River Basin (upper)	Y
		San Mateo Creek Basin	Y
		San Onofre Creek Basin	Y
		Santa Ana River Basin (lower)	N
		Santa Ana River Basin (upper)	N
		Santa Margarita River Basin (upper)	Y
		Santa Margarita River Basin (lower)	Y
		Santa Ysabel Creek Basin (lower)	Y
		Santa Ysabel Creek Basin (upper)	Y
		Sweetwater River Basin (lower)	Y
		Sweetwater River Basin (upper)	Y
Desert	2	Antelope‐Fremont River Basin	N
		Mojave River Basin	Y

^a^ Y = yes, N = no.

Our review of published and gray literature, and unpublished data, did not uncover any localized novel impacts that might suggest causes for a population crash or extirpation (besides the known drought). Potential threats such as OHV use, hiking, camping, bathing, trash, and exotic species were recorded at nearly all sites and all years surveyed. Also, several sites with known toad populations had been closed to public use for a prolonged period to protect the species from direct anthropogenic impacts (USFS, personal communication). We did not quantify prevalence of disturbances or collect data on disturbances over time.

**FIGURE 3:**
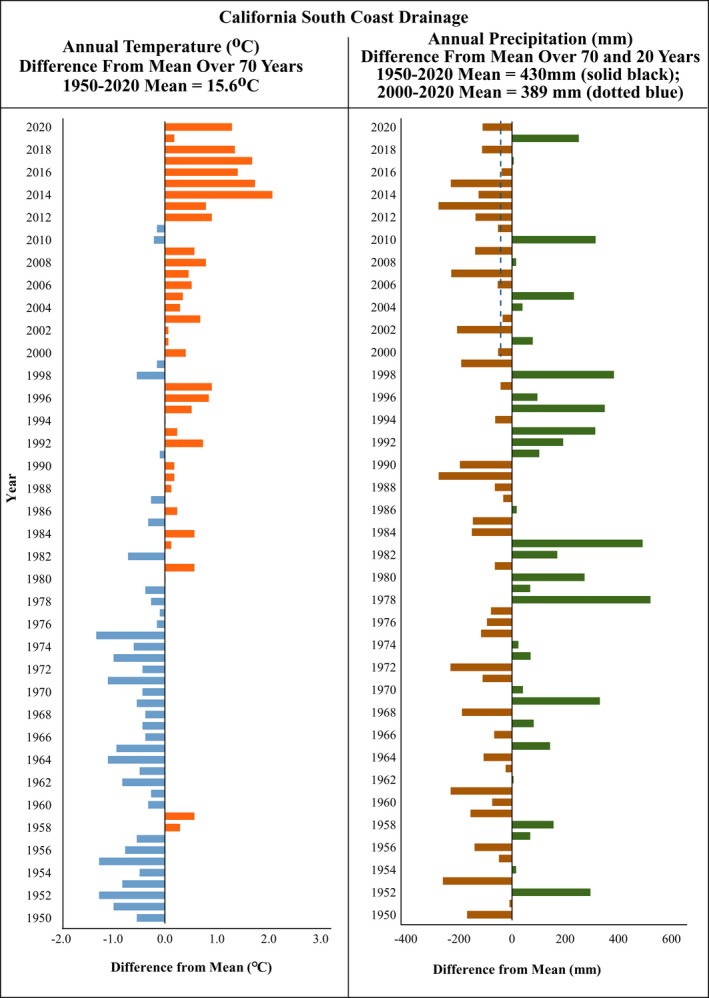
California South Coast Drainage annual temperatures (left) and precipitation (right) showing difference from mean over time. Mean precipitation has declined 40 mm in the last 20 years. Data are from NOAA (2022)

Our graph of annual temperature data for California's South Coast Drainage is consistent with LaDochy et al. (2007) in that average annual temperatures between 1950 and 2020 increased almost 2°C during this span of time (Figure 3), which is greater than what is reported for the state of California (increased 0.99°C since 1950; LaDochy et al., 2007). Average annual precipitation for southern California was highly variable from year to year but was 41 mm lower for the last 20 years compared to the last 70 years; between 1955 and 2020 the average annual precipitation was 430 mm whereas between 2000 and 2020 the average annual precipitation was 389 mm (Figure 3; https://www.ncdc.noaa.gov/cag/divisional/time‐series/0406/pcp/12/12/1950‐2021?trend=true&trend_base=10&begtrendyear=1950&endtrendyear=2022, accessed March 2022; NOAA, 2022).

## 4 DISCUSSION

We compiled results for 2017–2020 arroyo toad surveys conducted at all historical watersheds and most of the historical sites, hypothesizing that we would document numerous extirpations due to prolonged periods of drought. Given the lifespan of the toad (average 7–8 years; Fisher et al., 2018), the prolonged duration of the drought, and the comparison to extant sites from 1999 (20+ year duration), we expected to find fewer extant populations than we did. The short duration of time for our surveys (2017–2020) could have also produced an underestimation the number of extant populations. Toads were not detected at about 31% of the sites surveyed. Our data show that over the past 20+ years, this species has persisted in ~80% of the watersheds and ~69% of the sites surveyed, but has possibly disappeared from ~31% of these locations. Though this may imply arroyo toad persistence at the majority (~69%) of sites, we do not know if arroyo toad populations are stable or self‐sustaining at these sites, which is one of the Recovery Plan metrics (see USFWS, 1999, p. 76). Additional surveys are needed to determine if these extant populations are declining or at risk of extirpation. Overall, we consider the 61 extant sites to be a minimum estimate of extant populations because we did not survey 27 of 115 sites and because it is possible to have missed detection at some sites.

Though this species is known to be adapted to the generally hot, dry climate of southern California, increased drought severity and length may eventually surpass the limits of this species’ tolerance. Toads are more terrestrial than frogs and are known to have physiological adaptations for water retention, such as storing water in their bladder or metabolically producing water from their diet (Bundy & Tracy, 1977; McClanahan & Baldwin, 1969). Schmajuk and Segura (1982) show that toads in the *Bufo boreas* group specifically store more water in their bladder when deprived of it, and Jørgensen (1994) reports that the common toad (*B*. *bufo*) can retain up to 20% of its mass as water in the bladder when water deprived. Therefore, the xeric‐adapted arroyo toad likely uses this strategy to retain water through prolonged drought. Furthermore, arroyo toads may benefit from moderate drought because suitable conditions for breeding and metamorphosis generally occur in the form of slow‐moving braided streams when water levels are low. Though without the typical cycle of flooding and scouring events, habitat that is ordinarily lightly or moderately vegetated can fill in with riparian vegetation—including both native species such as mulefat (*Baccharis salicifolia*), cattail (*Typha* spp.), willow (*Salix* spp.), and invasive species such as giant reed (*Arundo donax*)—which can overtake areas formerly suitable for arroyo toad breeding (Brehme et al., 2006; Griffin & Case, 2001). While arroyo toads have persisted at most sites despite variable precipitation, our inability to detect the species at approximately 27 sites at which they were previously found suggests that the species is likely continuing to decline. In addition, desiccated adults documented by telemetry during drought years (Gallegos, 2011–2013, 2016, unpublished data), suggest that estivating toads are not impervious to drought effects on soil moisture.

Negative impacts from recreation, non‐native species, and altered hydrological regimes were documented at several locations (Ervin et al., 2006; Madden‐Smith et al., 2003; Matsuda et al., 2018; Miller et al., 2012) and may exacerbate the environmental challenges being experienced by these toads. More data are needed to quantify threats such as OHVs, habitat conversions, hydrological changes from dams, disease (i.e., chytrid fungus (*Batrachochytrium dendrobatidis*); Sweet & Sullivan, 2005), and predation, competition, or habitat manipulation from non‐native species (Richmond et al., 2021). Anthropogenic threats may also impact other native species associated with arroyo toads; therefore, addressing these threats may be a tractable and effective way to protect a suite of native species. For example, arroyo toads share or have historically shared habitat with several native common or special status species including western toad (*Anaxyrus boreas*), two‐striped garter snake (*Thamnophis hammondii*), red‐sided gartersnake (*Thamnophis sirtalis infernalis*), Santa Ana sucker (*Catostomus santaanae*), unarmored threespine stickleback (*Gasterosteus aculeatus williamsoni*), California red‐legged frog (*Rana draytonii*), western spadefoot (*Spea hammondii*), and western pond turtle (*Actinemys pallida*) (Richmond et al., 2013, 2014; Richmond, Jacobs, et al., 2014; Sweet & Sullivan, 2005). Anthropogenic alteration of habitat for waterplay (e.g., damming to create pools) and releasing unwanted pets (e.g., turtles, aquarium fish) or game fish for fishing often makes areas incompatible for native species and can promote persistence of non‐native species (Miller et al., 2012). Introduced species such as bullfrogs (*Lithobates catesbeianus*), rainbow trout (*Oncorhynchus mykiss*), several centrarchid, cyprinid, and ictalurid fish species, sliders (*Trachemys* sp.), and crayfish (*Procambarus* sp.) prefer, tend to be found, and can persist in areas where the habitat has been altered to contain areas with deeper pools, sometimes via non‐indigenous beaver (*Castor canadesis*) (Fisher & Shaffer, 1996; Miller et al., 2012; Richmond et al., 2021; Riley et al., 2005). These introduced species can negatively impact native species via direct predation or competition for resources (Bucciarelli et al., 2014; Matsuda et al., 2018; Miller et al., 2012). Maintaining natural shallow braided aquatic systems with sandy substrates and periodic drying may prevent many invasive predatory species from establishing by eliminating the pooled areas in which they are able to persist (Miller et al., 2012). Unfortunately, shallow braided streams and terraces with sandy substrate are also favored as locations for OHV use, which can be especially damaging to toad populations during the breeding and post‐breeding season when eggs, larvae, and metamorphs are reliant on surface water (Ervin et al., 2006; Griffin & Case, 2001). OHV use can cause direct mortality by crushing individuals burrowed under the soil or have indirect effects by habitat modification (e.g., soil compaction), thus reducing or preventing friable sands in which they burrow (Griffin & Case, 2001; Sweet, 1992). Driving in creekbeds also causes the collapse of berms and flattening of sand bars, which can drain occupied pools in braided sections (Sweet pers obs.). OHV and other recreational activity (e.g., mountain biking or equestrian use) within active streams and pools can dislodge sediments and harm both eggs and larvae (Ervin et al., 2006; Griffin & Case, 2001). Overall, protecting arroyo toads and their habitat from anthropogenic impacts could also help protect a host of other native aquatic‐associated species in southern California.

Some of the recovery tasks in the 1999 Recovery Plan have been studied and addressed to varying degrees. These include developing management plans, developing protocols for monitoring and surveying, managing dam releases in some areas, active research on exotic species interactions, toad movements, habitat analyses, and surveying areas within the potential range of the species (Brehme et al., 2006; Ervin et al., 2006, 2013; Fisher et al., 2018; Gallegos, 2011–2013, 2016, unpublished data; Madden‐Smith et al., 2003; Matsuda et al., 2018; Miller et al., 2012; Ramirez, 2003). This study contributes to the recovery tasks by providing the most comprehensive and up‐to‐date information on extant arroyo toad populations throughout their range in the United States. However, this study also had several limitations. Trying to cover the entire United States range while collecting and reporting data in a consistent manner was challenging due to the engagement of so many participants. A more stringent study design with fewer participants may have increased consistency of data collection methods and allowed for more rigorous analyses on occupancy; however, with fewer participants we may not have been able to survey as many sites. Population trend data and multiple visits over multiple years to all sites could also have improved our ability to accurately determine occupancy over time and help investigate one of the Recovery Plan's metrics (to document self‐sustaining populations “…equal to 20% or more of the average number of breeding individuals in seven of ten years…” see USFWS, 1999, p. 76). However, our main objective was to try and detect arroyo toads at as many of the historical sites as possible to provide a comprehensive understanding of which populations were still on the landscape and provide a baseline for future studies. This study provides information on which sites still need to be verified for toad persistence, and it may help identify additional sites within the range of the arroyo toad that could be explored for yet‐unknown populations (another metric of the Recovery Plan; USFWS, 1999). By identifying currently occupied sites, the study also could lead to new assessments of management at those sites.

These baseline data documenting the current occupancy status of the species, which had not been explored comprehensively or consistently for the past 20+ years, may help managers understand the current recovery status of arroyo toad. Currently, the Recovery Plan states that 20 (actually 19, see Ervin et al., 2013) self‐sustaining populations at specific locations are required for downlisting consideration. Though our data show that 20 of the 25 delineated watersheds in the Recovery Plan currently have extant populations (Table 1) and 18 of the 19 specific sites named within these watersheds have verified toad populations, data to assess whether or not populations are self‐sustaining are lacking. Detailed spatial and demographic data are needed to understand whether the Recovery Plan's definition of “self‐sustaining” has been met. This study may also inform the USFWS’ recovery planning into the future, including the number of sites that might constitute recovery. Defining such sites could involve considering factors such as proximity to other sites and types of negative impacts that may need to be mitigated within specific locations. This may also involve conducting repeated surveys during optimal years at the 27 non‐detection sites and 27 sites not surveyed.

Our comprehensive surveys confirmed that toads are extant at ~69% of sites; toads were not detected at ~31% of sites. Detection at the majority of sites suggests that arroyo toads may be better evolutionarily suited to the effects of drought cycle changes than previously understood. However, we emphasize that any tolerance to drought is not well‐studied. We suggest that minimizing anthropogenic impacts (including introduced aquatic invasive species) to historically and currently occupied sites may be the most effective strategy for arroyo toad conservation; this approach can also have positive implications for native species sharing the same habitat. The results of this study can inform recovery planning for the arroyo toad.

## CONFLICT OF INTEREST

None.

## AUTHOR CONTRIBUTIONS

**Cynthia J. Hitchcock:** Data curation (supporting); Formal analysis (equal); Methodology (equal); Project administration (equal); Supervision (equal); Writing – original draft (lead); Writing – review & editing (lead). **Elizabeth A. Gallegos:** Conceptualization (equal); Data curation (lead); Formal analysis (equal); Methodology (equal); Project administration (equal); Supervision (equal); Writing – original draft (supporting); Writing – review & editing (supporting). **Adam R. Backlin:** Conceptualization (equal); Data curation (supporting); Project administration (supporting); Supervision (supporting); Writing – original draft (supporting); Writing – review & editing (supporting). **Russell Barabe:** Data curation (supporting); Investigation (supporting). **Peter H. Bloom:** Data curation (supporting); Investigation (supporting). **Kimberly Boss:** Data curation (supporting); Investigation (supporting). **Cheryl S. Brehme:** Conceptualization (equal); Data curation (supporting); Investigation (supporting); Writing – review & editing (supporting). **Christopher W. Brown:** Data curation (supporting); Investigation (supporting). **Denise R. Clark:** Data curation (supporting); Investigation (supporting). **Elizabeth R. Clark:** Data curation (supporting); Investigation (supporting). **Kevin Cooper:** Conceptualization (supporting); Data curation (supporting). **Julie Donnell:** Data curation (supporting); Investigation (supporting). **Edward Ervin:** Data curation (supporting); Investigation (supporting). **Peter Famorlaro:** Data curation (supporting); Writing – review & editing (supporting). **Kim M. Guilliam:** Data curation (supporting); Investigation (supporting). **Jaquelyn J. Hancock:** Data curation (supporting); Investigation (supporting). **Nicholas Hess:** Data curation (supporting); Investigation (supporting). **Steven Howard:** Data curation (supporting); Investigation (supporting). **Valerie Hubbartt:** Data curation (supporting); Investigation (supporting); Writing – review & editing (supporting). **Patrick Lieske:** Data curation (supporting); Investigation (supporting). **Robert Lovich:** Conceptualization (equal); Data curation (supporting); Investigation (supporting); Writing – review & editing (supporting). **Tritia Matsuda:** Data curation (supporting); Investigation (supporting). **Katherin Meyer‐Wilkins:** Data curation (supporting); Writing – review & editing (supporting). **Kamarul Muri:** Data curation (supporting); Investigation (supporting). **Barry Nerhus:** Data curation (supporting); Investigation (supporting). **Jeff Nordland:** Data curation (supporting); Investigation (supporting). **Brock Ortega:** Data curation (supporting); Investigation (supporting). **Robert Packard:** Data curation (supporting); Investigation (supporting). **Ruben Ramirez:** Data curation (supporting); Investigation (supporting). **Sam C. Stewart:** Data curation (supporting); Investigation (supporting). **Samuel Sweet:** Data curation (supporting); Investigation (supporting). **Manna Warburton:** Data curation (supporting); Investigation (supporting). **Jeffrey Wells:** Data curation (supporting); Investigation (supporting). **Ryan Winkleman:** Data curation (supporting); Investigation (supporting); Writing – review & editing (supporting). **Kirsten Winter:** Data curation (supporting); Investigation (supporting). **Brian Zitt:** Data curation (supporting); Investigation (supporting). **Robert N. Fisher:** Conceptualization (equal); Data curation (supporting); Formal analysis (supporting); Funding acquisition (lead); Investigation (supporting); Methodology (equal); Project administration (equal); Supervision (equal); Writing – original draft (supporting); Writing – review & editing (supporting).

## Data Availability

The data that support the findings of this study are available within this article (Table 1 and Appendix). Authors may be contacted if more information is needed.

## 1

**Table:**

Recovery Unit	Watersheds	Site Name/Description	Detected 2017, 18, 19, or 20 (Y, N, N/S^a^)	2017–2020 Surveyed by^b^	Age Class Observed between 2017–2020 (A, J, M, T, E^c^)	Reported as Extant in 1999 Recovery Plan (Y, N, N/A, UN, PE, PX^a^)	Date(s) Reported Extant
**Northern**	**1. Salinas River Basin** (Monterey and San Luis Obispo Counties)	Salinas River, near city of Santa Margarita	N/S	–	–	PX	1936
		San Antonio River, above Lake San Antonio	Y	DOD	A, E	Y	1996, 2005, 2008, 2017
	**2. Santa Maria River Basin** (Santa Barbara County)	Sisquoc River, from Manzana Creek junction to Sycamore Campground	Y	USFS	T	PE	1991, 1993–4, 1999, 2000, 2007, 2017
	**3. Santa Ynez River Basin** (Santa Barbara County)	Agua Caliente Creek, from confluence w/Santa Ynez River upstream 2.5km	Y	USGS	T	N/A	2020
		Indian Creek, from confluence w/ Mono Creek upstream 1.5km	Y	USGS	M, T	Y	1989, 1992, 1993, 1999, 2000, 2020
		Mono Creek, from confluence w/Santa Ynez River upstream 1.5km	Y	USGS	M, T	Y	1989, 1992, 1993, 1999, 2000, 2020
		Santa Ynez River (upper), above Gibraltor Reservoir in scattered locations along 13km	N/S	–	–	Y	1989, 1992–3, 1999, 2000
		Santa Ynez River, at confluence with Agua Caliente Creek	Y	USGS	A	N/A	2020
	**4. Santa Clara River Basin** (Ventura and Los Angeles Counties)	Agua Blanca Creek, from confluence w/Piru Creek upstream ~2km	Y	ESA	A, T	Y	1992, 1999, 2000, 2010–11, 2017, 2020
		Castaic Creek, at Basin 3 above Castaic Dam	N	USGS	–	Y	2009, 2011, 2020
		Castaic Creek, below Castaic Dam for 4km	N/S	–	–	Y	1992, 1996, 2001, 2009, 2011
		Castaic Creek, between power plant and Fish Canyon	Y	USGS	T	Y	1996, 2001, 2020
		Lion Creek, at old Lion Campground (closed), at confluence w/Santa Clara River	N/S	–	–	Y	2010, 2011
		Piru Creek (lower), Blue Point Campground upstream to Lower Piru Gorge	Y	ESA	T	Y	1992, 2000, 2010–11, 2017
		Piru Creek (upper), from headwaters of Pyramid Lake upstream to Bear Gulch	N/S	–	–	Y	1989–91, 1999
		Piru Creek (upper), near Hardluck Campground	Y	USGS	A, J, M, T	PE	2009, 2012, 2020
		San Francisquito Creek	N/S	–	–	N/A	1997
		Santa Clara River, Soledad Canyon, from Hwy 14 to Agua Dulce Road	N	ECORP	–	N/A	2001
	**4. Santa Clara River Basin** (Ventura and Los Angeles Counties)	Sespe Creek, at Beaver Campground downstream to a large pool	Y	ESA; USFS; R2 Resource Consultants Inc.; USGS	A, M, T, E	Y	2011, 2018, 2019, 2020
		Sespe Creek, from Hot Springs Canyon upstream to mouth of Tule Creek	N/S	–	–	Y	1980s−90s, 1999, 2000
	**5. Los Angeles River Basin** (Los Angeles County)	Alder Creek, ~150m upstream w/ confluence of Big Tujunga Creek	Y	IND	A	Y	1999, 2011, 2017, 2018
		Arroyo Seco, just above Devil's Gate Reservoir	N/S	–	–	Y	1996, 1997, 1998
		Big Tujunga Creek (upper), 3N27 crossing and upstream of Big Tujunga Reservoir	Y	PSOMAS	A	Y	2011, 2018
		Big Tujunga Creek, ~1km south of I−210 crossing	N	USGS	–	PX	1915–1954
		Big Tujunga Creek, from confluence w/Alder Creek downstream ~1km	Y	IND	A, T	Y	1999, 2001, 2011–12, 2017, 2019
		Lynx Gulch Creek	N/S	–	–	N/A	2011
**Southern**	**6. Lower Santa Ana River Basin (**Orange County)	Santiago Creek	N	ICF	–	PX	1974
		Silverado Creek	N	ICF	–	PX	70s, 80s, 1998, 2005, 2008–9
	**7. Upper Santa Ana River Basin** (San Bernardino County)	Cajon Wash	N	Endemic Environmental; USGS	–	N/A	2000, 2005, 2007
	**8. San Jacinto River Basin** (Riverside County)	Bautista Creek	Y	MSHCP	A, T, E	PE	2002–3, 2010, 2017
		San Jacinto River	N	MSHCP	N/A	PE	2000, 2017
	**9. San Juan Creek Basin (**Orange and Riverside Counties)	Bell Canyon, from confluence w/San Juan Creek to Crow Canyon	Y	Dudek	A (calling)	Y	1998, 2017
		San Juan Creek, from Antonio Parkway to San Juan Hot Springs	Y	Dudek; USFS; MSHCP	M, T, E	Y	1974, 1992, 2010, 2017
		Trabuco Creek	N	ICF	–	Y	1997
	**10. San Mateo Creek Basin** (Orange, Riverside, and San Diego Counties)	Cristianitos Creek	Y	USGS	A, M, T	Y	1995, 1998, 2001, 2005, 2010, 2017, 2020
		Gabino Creek	N/S	–	–	Y	1995, 1998, 2001, 2005, 2010
		La Paz Creek	N/S	–	–	Y	1995, 1998, 2001, 2005, 2010
	**10. San Mateo Creek Basin** (Orange, Riverside, and San Diego Counties)	Los Alamos Canyon Creek	Y	USFS	T	N/A	1991, 1998–9, 2005, 2010, 2017
		San Mateo Creek, from estuaries to northern border of Camp Pendleton	Y	USGS	A, M, T	Y	1991, 1999, 2005, 2010, 2017, 2020
		San Mateo Creek, mainstem	N/S	–	–	Y	1991, 1999, 2005, 2010
		Talega Creek	Y	USGS	T	Y	1995, 1998, 2001, 2005, 2010, 2017, 2019, 2020
	**11. San Onofre Creek Basin** (San Diego County)	San Onofre Creek, from mouth to confluence of North and South Forks San Onofre Canyon	Y	USGS	A, M, T	Y	2010, 2017, 2020
	**12. Lower Santa Margarita River Basin** (San Diego County)	DeLuz Creek	Y	USGS	M, T	PE	2010, 2017, 2020
		Roblar Creek	Y	USGS	T	PE	2010, 2017, 2019
		Sandia Creek	N	USGS	–	PE	N/A
		Santa Margarita River, from the airfield to Fallbrook	Y	USGS; DOD	A, M, T	PE	2010, 2017, 2020
	**13. Upper Santa Margarita River Basin** (Riverside County)	Arroyo Seco Creek, Dripping Springs Campground	Y	USFS; IND	A, M, T, E	PE	1993, 2000, 2010, 2017, 2020
		Temecula Creek	N	IND	–	PE	1992, 2001, 2003, 2004
		Wilson Creek	N	IND	–	N/A	1998
	**14. Murrieta Creek Basin** (Riverside County)	Cole Creek	N	IND; USGS	N/A	N/A	2001, 2005
	**15. Lower and Middle San Luis Rey River Basin** (San Diego County)	Keys Creek	N/S	–	–	N/A	1998, 1999, 2001
		Pala Creek	N/S	–	–	Y	1959, 1998
		San Luis Rey River (lower), west of I−15	N	IND; USGS	–	Y	1991–2, 2010
		San Luis Rey River (middle), east of I−15	N	IND; USGS	–	Y	1928, 1996, 1998, 2000, 2004, 2011
	**16. Upper San Luis Rey River Basin** (San Diego County)	Agua Caliente Creek	Y	USFS; USGS	T	Y	1992, 1999, 2005, 2017, 2020
		Cañada Aguanga, ~2km upstream of confluence with San Luis Rey River	Y	USFS; USGS	T	N/A	1989, 1991, 2003, 2006, 2010, 2017
		San Luis Rey River (upper), Indian Flats	Y	USFS; USGS	T	Y	1932, 2017, 2020
		San Luis Rey River, above Lake Henshaw	Y	IND; USGS	A, M, T	Y	2019, 2020
	**16. Upper San Luis Rey River Basin** (San Diego County)	San Luis Rey River, West Fork	Y	USGS; IND	A, M, T	Y	1991–2, 1999, 2010, 2017, 2020
	**17. Lower Santa Ysabel Creek Basin** (San Diego County)	Boden Canyon, up to 3km north of confluence with Santa Ysabel Creek	N	USGS	–	N/A	2003–4, 2017
		Guejito Creek	N/S	–	–	Y	1937, 2005–2008
		San Dieguito River (upper), above Lake Hodges	Y	USGS	A, M, T	PE	2005, 2012, 2017, 2020
		Santa Maria Creek (lower), from confluence of Santa Ysabel Creek upstream 3km	N	USGS	–	PE	2001, 2005, 2008, 2010, 2016
		Santa Maria Creek (middle), near gaging station (5km upstream of confluence with Santa Ysabel Creek)	Y	USGS; IND	A, J, M, T	PE	2001, 2005, 2008, 2017, 2020
		Santa Ysabel Creek, at confluence with Boden Canyon	Y	USGS; CDFW; Merkel & Assoc.	A, M, T	N/A	2003–4, 2017, 2019, 2020
		Santa Ysabel Creek, at confluence with Temescal Creek	Y	USGS	T	PE	1996, 2020
		Santa Ysabel Creek, between Boden and Tim's Canyon	Y	USGS; CDFW; Merkel & Assoc.	A, M, T	N/A	2005, 2012, 2017, 2019, 2020
		Santa Ysabel Creek, between Sutherland Lake and Pamo Road	N/S	–	–	PE	–
		Santa Ysabel Creek, between Temescal Creek and Boden Canyon	Y	USFS; USGS; CDFW; Merkel & Assoc.	J, M, T	N/A	1991, 2005, 2008, 2017, 2020
		Santa Ysabel Creek, near confluence with Santa Maria Creek	Y	USGS	A	N/A	2017, 2020
		Temescal Creek, in Pamo Valley	Y	USFS; USGS	A, T	PE	1937, 1993, 2012, 2017, 2020
	**18. Upper Santa Ysabel Creek Basin** (San Diego County)	Santa Ysabel Creek and Tributary, west of Santa Ysabel Open Space Preserve	Y	USGS	M	N/A	2010, 2020
		Santa Ysabel Creek, above Sutherland Lake	Y	USGS	T	PE	2020
		Santa Ysabel Creek, at Witch Creek	N/S	–	–	PE	1991, 2005, 2008
	**19. Upper San Diego River Basin** (San Diego County)	Boulder Creek (lower)	N/S	–	–	N/A	N/A
		Cedar Creek, below falls	Y	USFS; USGS; Dudek	M, T	Y	2017
		San Diego River, below El Capitan Reservoir	N/S	–	–	Y	1993, 1997, 2002, 2008, 2016
	**19. Upper San Diego River Basin (**San Diego County)	San Diego River, between El Capitan Reservoir and Temescal Creek	Y	USFS; USGS; Dudek	A, M, T	Y	1993, 1997, 2002, 2008, 2017, 2020
		San Vicente Creek, ~1km south of Poole Ranch	Y	USGS; IND	T	Y	2017, 2020
		San Vicente Creek, West Branch	Y	IND	A	Y	1992, 1997, 2008, 2017, 2018
	**20. Lower Sweetwater River Basin** (San Diego County)	Sweetwater River (lower), Sycuan Peak Ecological Reserve	Y	USGS	J	Y	1999, 2000–1, 2005, 2008, 2010, 2017, 2019
		Sweetwater River, Sloane Canyon	Y	USGS; Sweetwater Authority	A, M, T	Y	2000–1, 2005, 2008, 2010, 2017
	**21. Upper Sweetwater River Basin** (San Diego County)	Peterson Creek/Canyon	Y	Sweetwater Authority	M, T	Y	1998–9, 2017
		Sweetwater River, above Hwy 79, Green Valley	N	USGS	–	Y	1990s, 2000–1, 2005, 2008, 2010
		Sweetwater River, along Merigan Fire Road	N	USGS	–	Y	1990s, 2000–1, 2005, 2008–9, 2010, 2012
		Sweetwater River, at Hulburd Grove	N/S	–	–	Y	1990s, 2000–1, 2005, 2008, 2010
		Sweetwater River, below Descanso Junction	N/S	–	–	Y	1990s, 2000–1, 2005, 2008, 2010
		Viejas Creek	N/S	–	–	UN	1996
		Viejas Creek, east of Alpine, near I−8 bridge	N	USFS	–	Y	1996
	**22. Lower Cottonwood Creek Basin** (San Diego County, Baja Caifornia, México ‐ not surveyed)	Campo Creek	N	USGS	–	PE	1923, 2008
		Cottonwood Creek (lower), below Barrett Reservoir	N	USGS	–	PE	1998, 2002–3, 2008, 2015
		Cottonwood Creek (lower), near Marron Valley	Y	USGS	A	Y	1998, 2002, 2003, 2008, 2017, 2020
		Potrero Creek	N	Merkel & Assoc.	–	PE	1923, 2010
		Tijuana River	N/S	–	–	UN	1998
	**23. Upper Cottonwood Creek Basin** (San Diego County)	Corral Creek	Y	USFS	M, T	N/A	2017
		Cottonwood Creek (upper), above Lake Morena	Y	Merkel & Assoc.; USFS	A, T	Y	1990–2, 1999, 2005, 2011, 2017, 2020
		Horsethief Canyon, for 2km (+) above Pine Valley Creek	Y	USFS	M, T	Y	1923, 1992, 2000, 2001, 2010, 2017
	**23. Upper Cottonwood Creek Basin** (San Diego County)	Kitchen Creek	Y	USFS; Merkel & Assoc.	T	PE	1923, 1990–2, 1999, 2005, 2011, 2017
		La Posta Creek	N	USFS	–	N/A	2005
		Miller Creek, Clover Flat	N	Merkel & Assoc.	–	N/A	–
		Morena Creek	Y	Merkel & Assoc.; USFS	A, T	PE	1923, 1993, 1999, 2017
		Noble Canyon	Y	USFS	A, M, T	PE	2017
		Pine Valley Creek	Y	USFS; USGS	A, M, T	Y	1923, 1991–2, 1998, 1999, 2001, 2009, 2017, 2020
		Pine Valley Creek, between Barrett Lake and Horsethief Canyon	Y	USFS	M, T	PE	1923, 1992, 2000–1, 2010, 2017
		Scove Canyon and Tributary	N	USFS	–	PE	1923
**Desert**	**24. Antelope‐ Fremont River Basin** (Los Angeles County)	Little Rock Creek, from the reservoir upstream	N	IND; USGS	–	Y	1996, 2001, 2011
		Santiago Canyon	N	USGS	–	N/A	1999, 2010
	**25. Mojave River Basin** (San Bernardino County)	Deep Creek, Devil's Hole	N/S	–	–	Y	1999, 2003
		Deep Creek, Hot Springs	N/S	–	–	Y	1999, 2005
		Grass Valley Creek	N/S	–	–	Y	1999, 2005–6
		Horsethief Creek, Summit Valley	Y	Endemic Environmental	M, T	N/A	2017
		Little Horsethief Canyon	Y	USFS	A	Y	1999, 2004, 2007, 2017
		Miller Canyon	N/S	–	–	Y	1999 ‐ common since 1930
		Mojave River (West Fork), Lake Silverwood	Y	Endemic Environmental	M, T	Y	1999, 2002–3, 2006–7, 2010, 2013, 2017
		Mojave River, vicinity of Mojave Forks Dam, in Mojave River, West Fork and Deep Creek	Y	USGS	A, J, M, T, E	Y	1999, 2001, 2008, 2010, 2020

^a^ Y, N, N/A, N/S, UN, PE, PX: Y = yes, N = no, N/A = no data available, N/S = not surveyed for the study, UN = unknown, PE = presumed extant, PX = presumed extinct, – = not observed.

^b^ Acronyms: USGS = U.S. Geological Survey, USFS = U.S. Forest Service, DOD = Department of Defense, CDFW = California Department of Fish and Wildlife, IND = independent consultant or researcher, ESA = Environmental Science Associates, ECORP = ECORP Consulting, Inc., ICF = ICF International, Inc., MSHCP = Multiple Species Habitat Conservation Plan.

^c^ A, J, M,T, E: A = adult, J = juvenile, M = metamorph, T = tadpole, E = egg string.
